# PEPICO analysis of catalytic reactor effluents towards quantitative isomer discrimination: DME conversion over a ZSM-5 zeolite

**DOI:** 10.1107/S1600577524004405

**Published:** 2024-06-25

**Authors:** Morsal Babayan, Evgeniy Redekop, Esko Kokkonen, Unni Olsbye, Marko Huttula, Samuli Urpelainen

**Affiliations:** a Nano and Molecular Systems Research Unit University of Oulu Oulu Finland; b Department of Chemistry, Centre for Materials Science and Nanotechnology (SMN) University of Oslo Oslo Norway; c MAX IV Laboratory Lund University Lund Sweden; Advanced Photon Source, USA

**Keywords:** PEPICO, zeolite catalyst, isomer selectivity, dimethyl ether to hydrocarbons conversion, reactor effluent analysis, synchrotron radiation, electron spectroscopy, ion mass spectrometry

## Abstract

The methanol-to-hydrocarbons (MTH) process involves the conversion of methanol, a C1 feedstock that can be produced from green sources, into hydrocarbons using shape-selective microporous acidic catalysts – zeolite and zeotypes. This reaction yields a complex mixture of species, some of which are highly reactive and/or present in several isomeric forms, posing significant challenges for effluent analysis. Conventional gas-phase chromatography (GC) is typically employed for the analysis of reaction products in laboratory flow reactors. However, GC is not suitable for the detection of highly reactive intermediates such as ketene or formaldehyde and is not suitable for kinetic studies under well defined low pressure conditions. Photoelectron–photoion coincidence (PEPICO) spectroscopy has emerged as a powerful analytical tool for unraveling complex compositions of catalytic effluents, but its availability is limited to a handful of facilities worldwide. Herein, PEPICO analysis of catalytic reactor effluents has been implemented at the FinEstBeAMS beamline of MAX IV Laboratory. The conversion of dimethyl ether (DME) on a zeolite catalyst (ZSM-5-MFI27) is used as a prototypical model reaction producing a wide distribution of hydrocarbon products. Since in zeolites methanol is quickly equilibrated with DME, this reaction can be used to probe vast sub-networks of the full MTH process, while eliminating or at least slowing down methanol-induced secondary reactions and catalyst deactivation. Quantitative discrimination of xylene isomers in the effluent stream is achieved by deconvoluting the coincidence photoelectron spectra.

## Abstract 2

Photoelectron–photoion coincidence (PEPICO) spectroscopy is used to analyse the composition of the effluent stream during dimethyl ether conversion on a ZSM-5 zeolite catalyst – a prototypical model reaction for the methanol-to-hydrocarbons process. Under well-defined low pressure (10^−6^ mbar) conditions, PEPICO offers a suitable analytical tool to identify the effluent composition, including quantitative isomer discrimination (in this case, of xylenes).

## 1. Introduction

Catalytic transformations of hydrocarbons and other organic molecules inside microporous acidic zeolites drive many large-scale industrial chemical processes with enormous combined economic and environmental impact (Cejka *et al.*, 2010 ▸). Some zeolite-mediated catalytic processes are also key candidates for implementing more sustainable transformations of diversified raw materials and renewable energy into vital energy carriers and chemical intermediates (Li & Yu, 2021 ▸). Highly convoluted reaction networks and microporous transport phenomena involved in catalysis by zeolites often present formidable experimental challenges. Complex effluent streams produced by zeolite catalysts may contain dozens of compounds, many of them present in several isomeric forms. Gas chromatography (GC) is a commonly used technique for effluent analysis in laboratory experiments that are typically conducted at ambient or above-ambient pressure conditions. However, GC is limited to the analysis of gas samples extracted from ambient or near-ambient pressure conditions and is not suitable for capturing highly reactive closed and open shell gaseous intermediates, *e.g.* formaldehyde, ketenes or radicals. Kinetic measurements under well defined, low pressure reaction conditions have emerged as an important source of mechanistic information because these highly reactive intermediates can be more readily detected and quantified by mass spectrometry (Brogaard *et al.*, 2014 ▸; Batchu *et al.*, 2017 ▸; Omojola *et al.*, 2021 ▸). Likewise, low pressure operation prolongs the catalyst lifetime by minimizing secondary reactions and coking, thus expanding the range of catalyst states that are amenable for precise kinetic characterization (Redekop *et al.*, 2020 ▸). Isomer-selective analytics at low-pressure conditions would open new avenues for advanced kinetic characterization of catalytic reactions at model operating conditions that facilitate in-depth mechanistic studies.

Photoionization mass spectrometry (PIMS) provides a suitable analytical platform for advanced mechanistic investigations, whereby an analyte is ionized by an incident photon and the resulting photoions are detected. PIMS achieves it’s full analytical potential when the energy of the incident light can be varied in the vacuum ultraviolet (VUV) range (6–42 eV), which typically requires a synchrotron radiation source. In comparison with conventional electron ionization mass spectrometry and gas chromatography, PIMS-based effluent analysis offers unique analytical advantages, including (i) better sensitivity and resolving power even for highly reactive species, (ii) applicability across a broad range of operating conditions, from vacuum to ambient pressure (with differential pumping), and (iii) in many cases, when performed with high photon energy resolution, offering isomer selectivity, which is particularly valuable for organic reactions. However, in order to gain a better insight into the gaseous composition, molecular photofragmentation must be considered in considerable depth (Kooser *et al.*, 2020 ▸). Although the electron and ion spectroscopy methods alone play a major part in studying molecular photofragmentation, in particular, recording and analyzing electrons and ions originating from the same photoionization event can lead to a more complete understanding of the photofragmentation process and the composition of the effluent gas stream. Taking the type of detected particles into account, coincidence techniques may be classified as photoelectron–photoion coincidence (PEPICO), photoion–photoion coincidence (PIPICO), photoelectron–photoion–photoion coincidence (PEPIPICO), photoelectron–photoelectron coincidence and photoion–neutral coincidence (Arion & Hergenhahn, 2015 ▸). PEPICO and threshold PIMS have been most instrumental in unraveling the reaction mechanisms of methanol-to-hydrocarbons (MTH), catalytic pyrolysis and oxychlorination processes (Hemberger *et al.*, 2020 ▸). With the aid of *in situ* synchrotron radiation PIMS, Wen *et al.* (2020 ▸) have detected formaldehyde (HCHO), an active intermediate, during the MTH reaction over two catalysts. Recently, Cesarini *et al.* (2022 ▸) utilized *operando* PEPICO spectroscopy to investigate reaction pathways for MTH and MCTH (methyl chloride-to-hydrocarbons) over the H-ZSM-5 zeolite catalyst. Using this technique they directly observed short-lived active intermediates, such as ketene and methyl radicals. Despite the increasing interest in applying these methods to investigate catalytic reactions, few experimental setups exist which combine catalytic microreactors with PIMS-based effluent analysis.

Herein, we report the establishment of such an instrument at the gas-phase endstation of the FinEstBeAMS beamline of MAX IV Laboratory (Lund, Sweden). Currently there are only a handful of synchrotron beamlines providing capabilities for the PIMS-based analysis of catalytic reactor effluents, including FinEstBeAMS. Features of some of them are are compared in Table 1 ▸. The main differences between these setups are the accessible energy ranges and the types of available detectors. The VUV beamline at the Swiss Light Source (SLS) offers photons in the 3–150 eV range and is capable of both photoion and photoelectron analysis (SLS, 2023 ▸). The BL03U beamline at the National Synchrotron Radiation Laboratory (NSRL) in China has a relatively narrow photon energy range of 5–21 eV and only detects ions (Zhou *et al.*, 2016 ▸). The DESIRS beamline at Synchrotron SOLEIL, France, covering the VUV range (5–40 eV) is equipped with a double imaging photoelectron photoion coincidence (i^2^PEPICO) spectrometer with two position-sensitive detectors to detect both photoelectrons and photoions (Tang *et al.*, 2015 ▸). In comparison, FinEstBeMS covers a broad range of photon energies, 4.4–1000 eV, which enables both valence and core ionization, and features ion and electron analysis as well as coincidence experiments.

The conversion of methanol and/or dimethyl ether (DME) to hydrocarbons, abbreviated as MTH and DTH, respectively, on acidic zeolites and zeotypes offers a prototypical example of a catalytic reaction which produces compositionally and isomerically complex effluents (Olsbye *et al.*, 2012 ▸). MTH/DTH are promising industrial routes towards hydrocarbon fuels and platform chemicals, which can accommodate diverse feedstocks including bio-gas and captured CO_2_ (Xie & Olsbye, 2023 ▸). In acidic zeolite or zeotype catalysts, an equilibrated mixture of DME and methanol react on Brønsted acid sites (BAS) to produce larger hydrocarbon molecules including alkenes, alkanes and aromatics. The reaction proceeds through the dual-cycle hydrocarbon pool (HCP) mechanism in which several pathways can be distinguished. The first C—C bonds are formed from DME and/or methanol via surface methoxy species (SMS) and highly reactive intermediates such as formaldehyde or ketene, eventually leading to C2–C3 alkenes. These primary products are then repeatedly methylated by SMS (stepwise pathway) or gaseous DME/methanol to form C3–C5+ alkenes – the so-called alkene cycle. The product distribution is further controlled by co-occurring cracking reactions. Hydrogen transfer reactions between methanol and alkenes, also catalyzed by BAS, lead to the formation of alkanes and aromatics. The latter can sustain an independent cycle of sequential methylation and cracking – the aromatics cycle. At steady state, the population of *in situ* generated alkene and aromatics intermediates termed the HCP resides within the catalyst and mediates continuous catalytic production of products from DME/methanol, in parallel with the hydrogen transfer and isomerization reactions. Moreover, methyl radicals were also detected in the reaction medium, suggesting that the underlying chemistry may be even more complex than previously thought. Eventually, the growth of large polyaromatic molecules and coke occludes the microporous space and deactivates the catalyst.

Catalytic performance, *i.e.* activity, selectivity and stability, is ultimately controlled by a multitude of factors related to the catalyst structure and operating conditions. In order to establish the structure–performance relationships and optimize the catalytic materials and reactions, it is imperative to better understand the kinetics of different reaction pathways and how they are affected by variations of the materials composition and structure. However, disentangling the distinct reaction pathways and individual reaction steps is a challenging experimental task, given the aforementioned complexity. PIMS-based methodologies for the reaction analysis have already provided essential mechanistic insights into methanol-to-olefins (MTO) chemistry, and this provides the main motivation for the current study – to showcase the new *operando* PEPICO capabilities at the FinEstBeAMS beamline in the context of this important reaction. In particular, we demonstrate PEPICO analysis of DME conversion on a ZSM-5 catalyst with a particular emphasis on quantitative isomer discrimination for the product xylenes.

## 2. Experiments

In this study, the products of the DME reaction over the ZSM-5 zeolite catalyst at a temperature of 375°C are investigated in real time, during the catalytic process, through *operando* PEPICO spectroscopy. This investigation has been performed using a coincidence setup at the gas-phase endstation (GPES) of the FinEstBeAMS beamline at the MAX IV Laboratory. Pure DME was fed into the reactor at a rate of 0.045 s.c.c.m. (standard cubic centimeters per minute) via a precision leak valve opening directly into the packed bed. At this feed rate, the pressure in the analysis chamber remained at 5 × 10^−7^ mbar. In this experiment, photons with an energy of 40 eV were utilized as the ionization source. The electron analyzer operates at a pass energy of 100 eV with the kinetic energy window centered at 28 eV. Hence, electrons with kinetic energies within the range 23–33 eV (equivalent to binding energies of 7–17 eV) were captured. Moreover, the measurement was conducted at the magic angle of 55°. While the beamline has a sub-meV resolution at 40 eV photon energy (Pärna *et al.*, 2017 ▸), the electron spectrometer resolution was approximately 600 meV. The electron count rate and random trigger frequency were kept at about 25 Hz to have a better coincidence purity. In total, over a duration of approximately 15 h, nearly 2 × 10^6^ triggers were detected, in which roughly 21% of them were coincidence triggers. Coincidence-specific data handling was performed with custom *Igor Pro* macros (Kukk *et al.*, 2007 ▸).

### 2.1. Gas-phase endstation

The FinEstBeAMS beamline is in the 1.5 GeV storage ring providing photon energy in the range from ultraviolet to soft X-ray (*i.e.* 4.5 to 1300 eV) as well as variable polarization of synchrotron radiation. Detailed information on the design and optical concept of the FinEstBeAMS beamline is given elsewhere (Pärna *et al.*, 2017 ▸; Chernenko *et al.*, 2021 ▸). The FinEstBeAMS beamline consists of two separate branch lines. The GPES and the photoluminescence endstation (PLES) are in the same branch, while the solid-state endstation (SSES) is located in the other one.

The GPES was developed for electron and ion spectroscopy as well as photoelectron–photoion coincidence spectroscopy of low-density matter. Kooser *et al.* (2020 ▸) describe the GPES in more details. Briefly, in this apparatus a modified hemispherical electron energy analyzer (SCIENTA R4000) equipped with a fast resistive anode position-sensitive detector is utilized to detect photoelectrons. Moreover, a Wiley–McLaren ion time-of-flight (TOF) mass spectrometer with delay-line position-sensitive detector detects ions produced throughout the ionization. In order to calibrate the TOF spectrum to masses, the following formula is utilized:*m*/*z* = (TOF − *T*_0_)^2^/*C*^2^. Here, the calibration parameters are determined by using the peaks corresponding to masses 2 (H_2_) and 91 (C_7_H_7_) as the initial guess. Using these initial values, we calculate *T*_0_ = 1715 ns and *C* = −11.59 ns (e/a.m.u.)^1/2^ (in which e and a.m.u. are the elementary charge and atomic mass units, respectively) which ensures that all other TOF peaks fall into the correct masses.

### 2.2. Reactor

**Figure 1:**
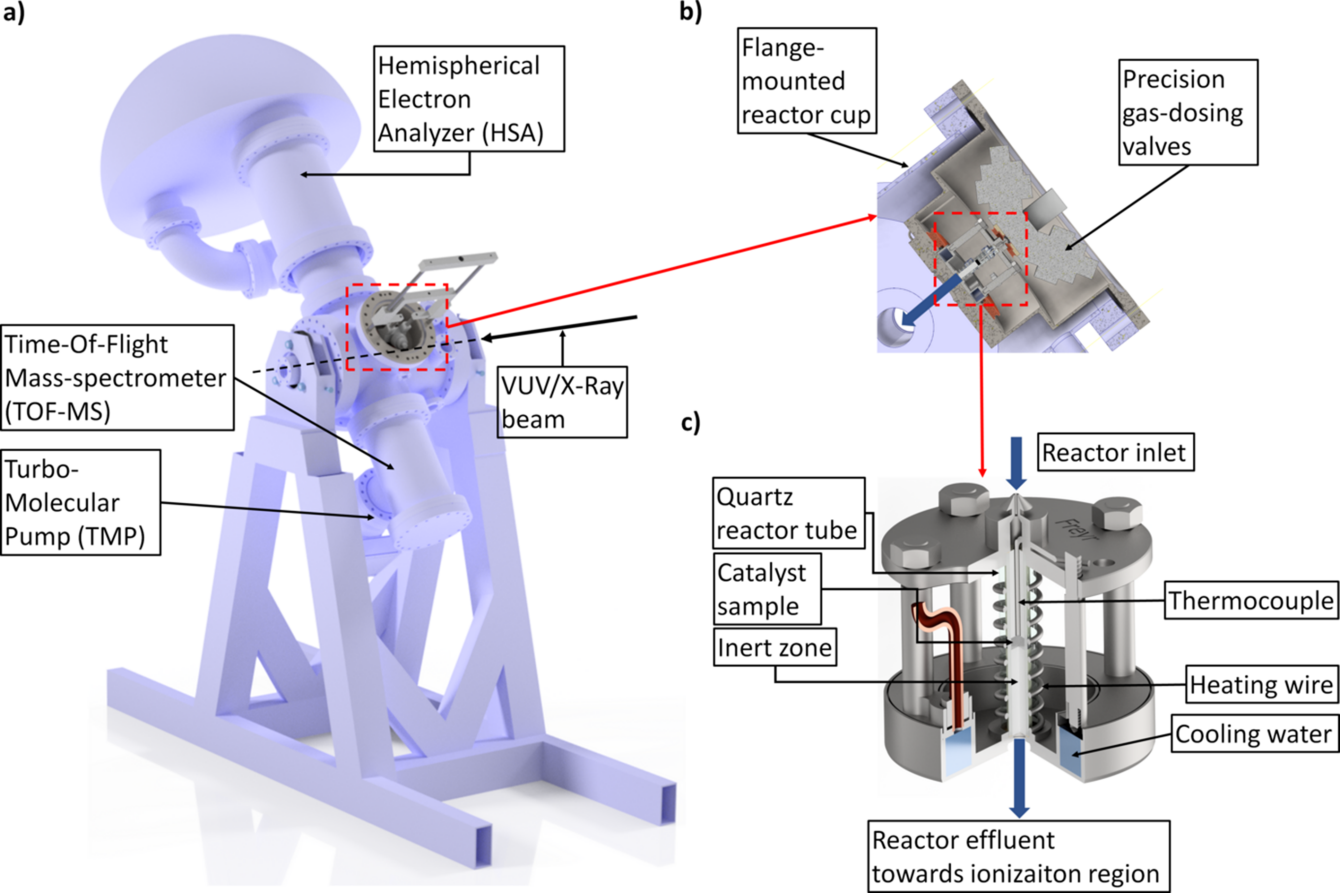
Schematic of the reactor connected to the gas-phase endstation at the FinEstBeAMS beamline of MAX IV Laboratory.

A portable, flange-mounted catalytic packed-bed reactor (length 50 mm, internal diameter 4 mm) was interfaced directly with the GPES endstation, as shown in Fig. 1 ▸. In brief, 20 mg of a catalytic sample was packed in the middle of the reactor as a thin (2 mm) layer sandwiched between two inert zones that were packed with quartz particles of the same sieve fraction. The reactor was resistively heated, while the temperature was monitored by a K-type thermocouple positioned in the middle of the catalytic bed. Gaseous reactants were fed into the reactor through a calibrated leak valve, and the effluent was allowed to freely enter the analysis chamber where the gas was pumped away by three turbomolecular pumps with total capacity of about 1300 l s^−1^. A schematic of the reactor connected to the GPES is presented in Fig. 1 ▸.

### 2.3. Materials and reagents

A commercial ZSM-5-MFI-27 zeolite was purchased from Sud Chemie. To obtain the acidic form of the catalyst, the as-received material was ion-exchanged with NH_4_NO_3_, extensively washed, and calcined in static air at 550°C for 10 h. Then, the catalyst was pressed into pellets that were sieved to 250 < *d*_p_ < 400 µm size fraction, which were subsequently packed into the reactor. Before the experiment, the catalyst was maintained in vacuum at 550°C for 30 min to desorb the residual water. A detailed procedure for the catalyst preparation and extensive standard characterization data has been given by Rojo-Gama *et al.* (2018 ▸).

Basic physico-chemical properties were determined to be as follows: Si/Al ratio of 15, BAS (Brønsted acid sites) concentration of 0.87 mmol g^−1^, crystal size of 2–6 µm, and BET (Brunauer–Emmett–Teller) surface area of 398 m^2^ g^−1^.

### 2.4. PEPICO

In this study, *operando* PEPICO spectroscopy is used, which, aside from being isomer-selective, is able to qualitatively differentiate short- and long-lived species as well.

In PEPICO spectroscopy, both the photoelectron and the photoion generated via the ionization are detected. For regular PEPICO spectroscopy, the kinetic energy of electrons in conjunction with cations with the same mass per charge ratio provides the mass-resolved photoelectron spectrum (ms-PES) for that specific cation. However, in threshold-PEPICO spectroscopy, photoions in coincidence with electrons having near-zero kinetic energy are collected. Therefore, a mass-selected threshold photoelectron spectrum (ms-TPES) is provided for a particular mass per charge ratio by threshold-PEPICO (Bodi *et al.*, 2013 ▸). To detect different isomers using threshold-PEPICO, tunable light sources with sufficiently high resolution are required. Hence, the regular PEPICO technique (from hereon PEPICO), readily available at FinEstBeAMS, is considered in this study.

The products of the reaction and the remaining reactant (when the conversion rate is less than 100%) leaving the microreactor are ionized by the photon beam. This leads to the generation of ions and electrons in the extraction region of the TOF mass spectrometer. When an electron is detected by the electron analyzer, a signal is generated to initiate a pulsed electric field in the extraction region of the mass spectrometer.

Since the electron mass is negligible compared with the ions, the flight times of electrons are significantly lower than for the ions, therefore it is reasonable to assume that the formation of the ions and the detection of the electron occur concomitantly. Eventually, the electron–ion pairs associated with the same photoionization event can be distinguished by correlating the detected electrons and ions.

#### 2.4.1. True/false coincidences

Although one electron is detected each time, ionizing more than one atom or molecule throughout the ionization process is possible which leads to the detection of electron–ion pairs that are not coming from the same event. Such events are known as false coincidences. By applying a low ionization rate, the probability of detecting the electron–ion pairs arising from the same event (true coincidences) increases, as the generated electron–ion pairs will be well separated in time (Bodi *et al.*, 2013 ▸). Furthermore, less than a 100% detection efficiency of the electron and ion detectors (which is lower for the electron) contributes to the false coincidences. To distinguish the ions generated by true coincidences, subtracting the random ions coming from the false coincidences is required. Consequently, a reference random coincidence must be measured under exactly the same conditions. Hence, an external pulse generator is used to create artificial random triggers besides electron triggers. For those random triggers, all measured coincidences would be false coincidences. Eventually, subtracting the random coincidences from total coincidences coming from electron triggers leads to true coincidences. Previously (Prümper & Ueda, 2007 ▸), coincidence experiments and the random coincidences subtraction method have been described in more detail. Here, random triggers generated by an external pulse generator with 25 Hz frequency is utilized. The TOF spectra from electron-triggered, random and true coincidences are depicted in Fig. S1 of the supporting information.

#### 2.4.2. Electron spectra

PEPICO measurements provide both the TOF of ions, revealing the mass per charge ratio of the ions, as well as the kinetic energy of the ejected electrons. This technique is commonly used to analyze the unimolecular dissociation. However, analyzing catalytic reactions often involves dealing with multiple products and leftover reactants, making it difficult to distinguish parent molecules from ionization fragments. In these cases, coincidence ion yield photoelectron spectra (CIY-PES), analogous to ms-PES referred to by Cesarini *et al.* (2022 ▸), can be used to provide additional information. For atoms, photoelectron spectroscopy shows the binding energies of the electrons, whereas, for molecules, it reveals vibrational and rotational excitations as well.

Photoelectron spectroscopy reveals key features of the original molecular orbitals from which the electrons are emitted. On the other hand, isomers of a molecule have distinct electronic structures due to differences in the relative positioning of substituent groups, such as methyl groups in xylene, which can alter electronic density and molecular symmetry. This makes photoelectron spectroscopy a useful tool not only for distinguishing between different molecules but also for identifying various isomers of a molecule. For example, the photoelectron spectra obtained for *m*- and *p*-xylene by Koenig *et al.* (1974 ▸) using He(I) radiation revealed that the first ionic states of these isomers have distinct vertical ionization potentials that can be utilized for differentiation purposes. Likewise, unique features observed in the photoelectron spectra of *cis*-, *trans*- and *iso*-butene can serve as distinctive markers to differentiate between these isomers (Ying *et al.*, 1993 ▸). Therefore, utilizing CIY-PES can be a valuable tool for differentiating between ions with identical mass/fragments.

## 3. Results and discussion

### 3.1. TOF spectra

The detected ions, representing the effluent stream during the conversion of DME on the ZSM-5 catalyst, are generated mainly by single valence ionization, as the cross section for direct double photoionization is low. Therefore, for the sake of abbreviation, instead of mass per charge ratio, only mass is going to be used.

**Figure 2:**
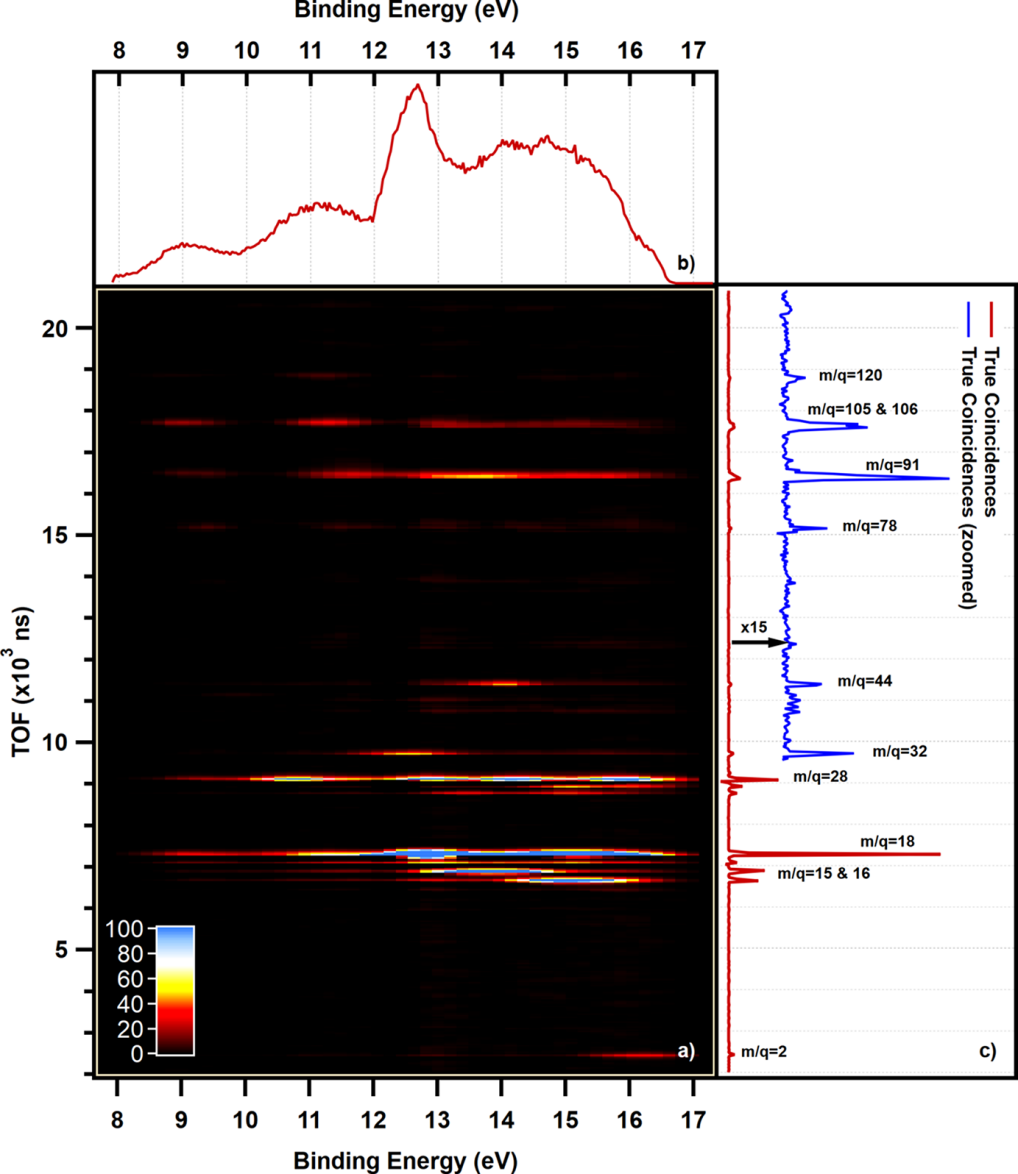
(*a*) PEPICO map of the effluent stream of DME conversion over ZSM-5 zeolite at 375°C ionized using light with 40 eV energy, (*b*) integrated binding energy spectrum and (*c*) integrated ion TOF spectrum with false coincidences subtracted. For higher TOF, the spectrum intensity is multiplied by 15 to make it more clear. The corresponding mass per charge ratios are mentioned for groups of TOF peaks.

A broad range of ions, covering masses from 1 to 156, are detected in the effluent stream. Table 2 ▸ lists the masses detected in true coincidences and the possible corresponding molecular ions. The calibrated TOF spectrum of true ions detected in coincidence with electrons in the binding energy range of approximately 8 to 17 eV is depicted in Fig. 2 ▸(*b*). Due to the negligible intensity observed for ions with masses greater than 120, they are excluded from the figure. Furthermore, it is noteworthy to highlight that the comparison between the TOF spectrum for DME flowing over the blank reactor closely resembles the reference spectrum provided in the *NIST Chemistry WebBook* (Wallace, 2018 ▸). This correspondence effectively mitigates concerns regarding the potential reactivity of DME (on the walls of the reactor and/or inert packing) under these experimental conditions. The TOF spectra of a mixture of Ar and DME (3:1 ratio) flowing over the empty reactor is plotted in Fig. S2 for two different rector temperatures.

In the current experiment, DME is not present in the effluent and its complete conversion is attained under the considered conditions. Feng *et al.* (2001 ▸) reported that, at 40 eV photon energy for DME, the branching ratios of ions with masses 46 and 45 were about 13.6% and 22.5%, respectively. However, ions with these masses are not detected in this study (as shown in Table 2 ▸).

In Fig. 2 ▸(*b*), the peak with the highest intensity corresponds to H_2_O^+^ (mass 18) – an expected result, considering that water is a major product of DME conversion on zeolite catalysts (Chang, 1983 ▸). The peak with the second highest intensity corresponds to the mass 28, which can be attributed to N_2_^+^, CO^+^ and/or C_2_H_4_^+^ cations. However, it is not possible to determine from the TOF spectrum alone whether all of these cations contribute to this peak or not. Moreover, the peaks for masses 16 and 15 exhibit noticeable intensities in the recorded spectrum. The CH_3_^+^ cation is the only plausible candidate for the peak with mass 15, given that the effluent is expected to contain hydrocarbons, water and residual oxygenates (DME and methanol). However, it is possible that both CH_4_^+^ and O^+^ contribute to the peak observed at the mass 16. Based on the peaks observed at masses 18, 28, 32 and 44, it is possible that the O^+^ cation detected in the experiment originates from the fragmentation of H_2_O, CO, O_2_, CH_3_OH or CO_2_ molecules, respectively. Nonetheless, the absence of a peak at mass 31 implies that CH_3_OH is not present in the effluent stream, which is consistent with a complete conversion of oxygenates on ZSM-5 catalyst at these conditions. For the peak at mass 44, both CO_2_^+^ and C_3_H_8_^+^ are the possible cations that we cannot discriminate only by TOF measurement. There is a group of small peaks at masses 39–42, which originate possibly from photofragmentation of propylene.

The TOF spectra show a pair of peaks at masses 91 and 92, which correspond to C_7_H_7_^+^ and C_7_H_8_^+^, respectively, and are the major fragments of toluene. Moreover, apart from C_8_H_9_^+^ and C_8_H_10_^+^, C_7_H_7_^+^ is a main fragment of xylene. The pair of peaks observed at 105 and 106 represent C_8_H_9_^+^ and C_8_H_10_^+^ cations, respectively. Although the TOF spectrum provides valuable information about the potential parent molecules in the effluent stream, toluene and xylene in this case, it is not possible to determine the specific origin of the C_7_H_7_^+^ cation.

In general, conventional mass spectroscopy techniques, including TOF, have limitations when it comes to analyzing the complex mixtures or molecules with identical masses (like isomers) or those exhibiting similar fragments. Hence, alternative techniques like PEPICO are employed to overcome these limitations and provide more detailed information about the parent molecules. This is achieved by recording the kinetic energy of the detected electrons and extracting CIY-PES for each detected masses. These spectra can then be compared with the previously reported spectra of potential parent molecules to validate the identification achieved by mass spectrometry.

### 3.2. PEPICO

Fig. 2 ▸ shows an electron-energy-resolved PEPICO map of electronic states with binding energies between 8 and 17 eV. This figure shows a map of the event intensities for all ion TOF and electron energy pairs. The horizontal axis corresponds to the electron hit position energies (which is calibrated to the electron binding energy in the top panel) and the vertical axis corresponds to the simultaneously detected ion flight times. This map provides an overview of the fragmentation patterns by associating specific electron binding energies with their corresponding positively charged fragments. False coincidences have been removed and slight smoothing is applied for better visualization. While the general trend can be seen in Fig. 2 ▸, a more detailed analysis should be conducted using the CIY-PES, which is given in the next section.

### 3.3. CIY photoelectron spectra

As described in Section 3.1, the TOF spectrum furnishes valuable insights into the mass distribution of ions within the chamber, along with their relative abundances, aiding in compound identification to a certain extent. However, when it comes to molecules with identical masses or a mixture of samples exhibiting similar ionization fragmentation patterns, TOF is limited. For instance, the mass 91 ionization fragment is predominant in both xylene and toluene, making them indistinguishable in a mixture using only TOF. CIY-PES within the framework of PEPICO addresses such challenges by providing additional information about the emitted photoelectrons in coincidence with the mass of interest.

Fig. 2 ▸(*a*) illustrates the photoelectron spectrum (PES) of all detected electrons in coincidence with true ions. In this section, we aim to discriminate between the different species in the effluent stream by comparing the extracted CIY-PES with those previously reported for potential source compounds. To perform this comparative analysis, reference spectra are digitized using the *WebPlotDigitizer* online software (Rohatgi, 2022 ▸).

The TOF spectrum reveals two intense peaks at masses 15 and 16 [Fig. 2 ▸(*b*)]. The peak at mass 15 corresponds only to the CH_3_^+^ cation, while that at mass 16 can be attributed to CH_4_^+^ and O^+^ cations. The potential parent molecules that contain oxygen and can give rise to the O^+^ ion in this study are O_2_, CO, CO_2_, water and DME. However, the mass spectra of these molecules, as reported in the *NIST Chemistry WebBook* (Wallace, 2018 ▸), would exhibit a considerably smaller peak at mass 16. In our dataset, DME undergoes complete conversion, and the molecular ions of O_2_, CO and CO_2_ have even lower intensities than mass 16. Additionally, according to the *NIST Chemistry WebBook*, the mass 16 fragment of water is negligible. Therefore, it is assumed that CH_4_^+^ is the main cation contributing to the peak in question.

**Figure 3:**
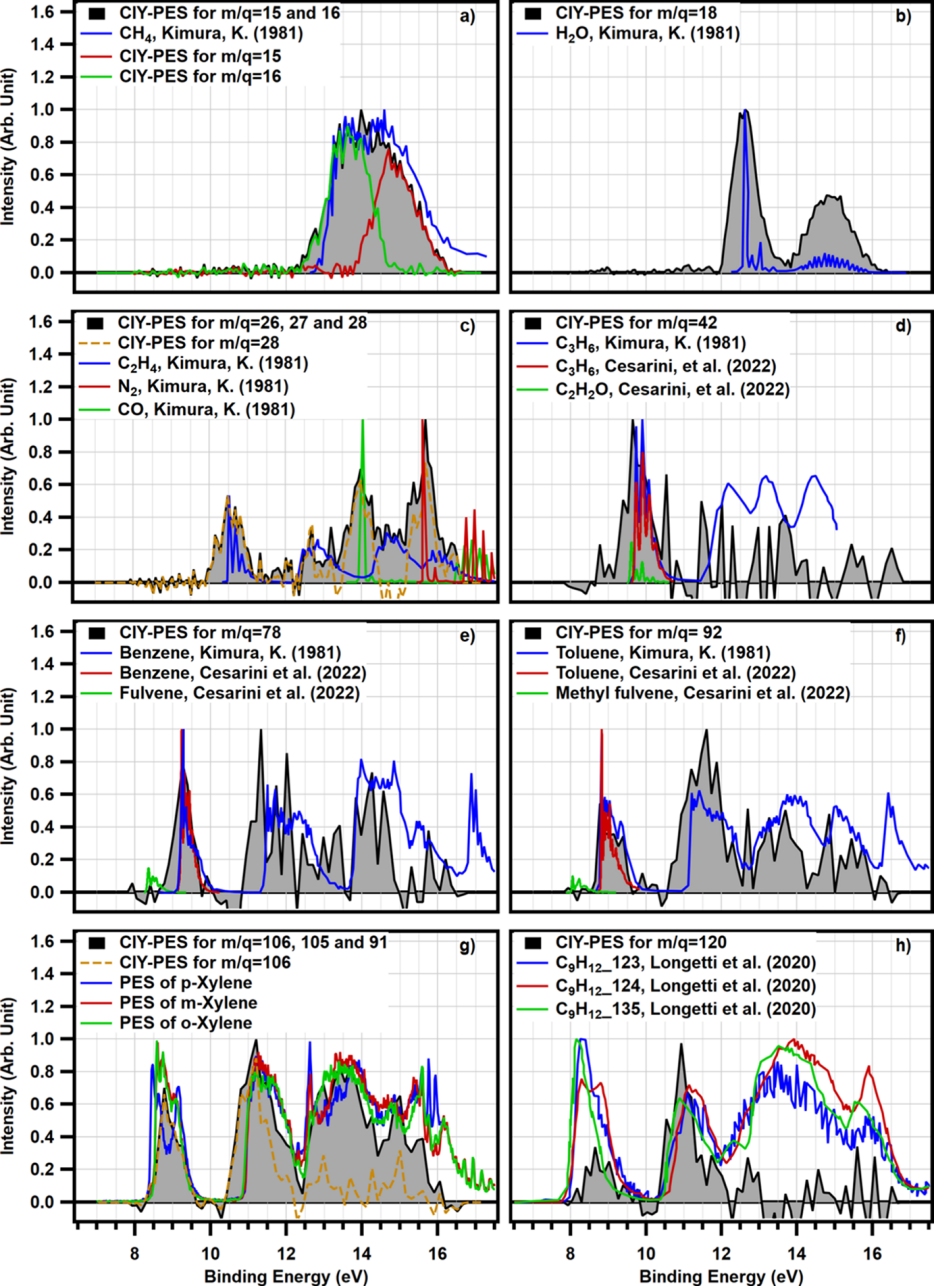
Comparison of extracted coincidence ion yield photoelectron spectra (CIY-PES) at 40 eV photon energy with reference spectra for the most intense detected cations (Kimura, 1981 ▸; Cesarini *et al.*, 2022 ▸; Koenig *et al.*, 1974 ▸; Longetti *et al.*, 2020 ▸).

Fig. 3 ▸(*a*) shows a comparison between the CIY-PES for masses 15 and 16, and their sum with the reference PES of CH_4_. The CIY-PES of mass 16 only covers a portion of the reference spectrum, while the CIY-PES of mass 15 covers the remaining section. The sum of these two spectra results in a spectrum that closely resembles the reference spectrum of CH_4_ as reported by Kimura (1981 ▸). The ionization of a molecule can occur from different sites, including the bonding and antibonding orbitals. For CH_4_, the main fragmentation channels are (Chang *et al.*, 2017 ▸)
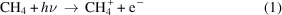

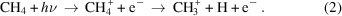
Therefore, aggregating the CIY-PESs collected with PEPICO for the main fragments (in this case CH_3_^+^ and CH_4_^+^) can result in a spectrum that is more representative of the reference spectra (*i.e.* CH_4_ PES). This is because, in reference spectra, electrons from all ionization channels, including all originating from both inner valence ionization and outermost shell ionization, are considered, whereas, in CIY-PES, only electrons generated in a specific ionization channel are taken into account.

Mass 18 exhibits the most prominent peak in the TOF spectrum, with water being the most likely species of origin, as stated previously. To verify this, the extracted CIY-PES for mass 18 is compared with the He(I) photoelectron spectrum of water in Fig. 3 ▸(*b*) (Kimura, 1981 ▸). Despite the differences in the photon energies and the resolution, this figure demonstrates a favorable agreement between the measured and the reference spectra.

Based on the nature of the experiment, the most likely cations with mass 28 are C_2_H_4_^+^ (a product of the reaction), CO^+^ (a chamber contaminant) and N_2_^+^ (an air leak). Comparison of the corresponding CIY-PES for mass 28 with the reference He(I) photoelectron spectra of N_2_, CO and C_2_H_2_ (Kimura, 1981 ▸) (see Fig. S4) reveals that N_2_ and CO are present in the effluent stream. However, the reference spectrum for C_2_H_4_ displays a broad peak at a binding energy of 14.66 eV which is not captured by only the CIY-PES for mass 28. According to previous studies (Wallace, 2018 ▸; Stockbauer & Inghram, 1975 ▸), in addition to the molecular ion C_2_H_4_^+^, pure C_2_H_4_ also fragments into C_2_H_3_^+^ and C_2_H_2_^+^ with masses 27 and 26, respectively. The combined CIY-PES for masses 26, 27 and 28, indeed, demonstrate a better agreement with the reference photoelectron spectrum of pure C_2_H_4_ because it includes all major fragmentation channels [as presented in Fig. 3 ▸(*c*)].

The detection of N_2_ indicates the presence of air leakage into the chamber, which is also confirmed by the observation of O_2_ with a distinct peak at mass 32 in the TOF spectrum [Fig. 2 ▸(*b*)] and the corresponding comparison of CIY-PES with the reference photoelectron spectrum for O_2_ (Kimura, 1981 ▸) (see Fig. S5).

Ions with masses of 41 and 42 are also detected, albeit with low intensity. These ions could correspond to propylene (C_3_H_6_), which is an expected product of DME conversion, and ketene (C_2_H_2_O), which is a highly reactive intermediate of the reaction. Due to moderate spectral resolution and low signal-to-noise ratio, it is not possible to distinguish them from the PES. However, it can be inferred that the signal corresponds to propylene, since ketene is so reactive that it is not expected to survive the transport through the second inert zone in the reactor. Furthermore, mass 14 is the predominant fragment of ketene (Wallace, 2018 ▸), which is not detected in our TOF spectrum [Fig. 2 ▸(*c*)]. The comparison between extracted CIY-PES for mass 42 and reference spectra for C_3_H_6_ and ketene is depicted in Fig. 3 ▸(*d*).

Ions with mass 44 likely originate from CO_2_ and propane C_3_H_8_, the latter being an expected minor product of DME conversion via hydrogen transfer reactions between propylene and methanol. However, CIY-PES of mass 44 (Fig. S6) reveals that C_3_H_8_ does not exist in the chamber and all ions with mass 44 are CO_2_^+^. We attribute the presence of CO_2_ to a combination of (i) the chamber background, (ii) the product of decarboxylation of MTH reaction intermediates on the zeolite (Huber & Plessow, 2023 ▸), as well as (iii) the product of oxidation of hydrocarbons by a minute amount of oxygen in the background (Fig. S5).

Ions with mass 56 can be assigned to butene – another expected product of the reaction formed by methylation of propylene. However, the production of butene was limited, leading to a low signal-to-noise ratio for CIY-PES of mass 56.

According to Cesarini *et al.* (2022 ▸), various isomers of C_5_H_8_ can undergo additional cyclization/dehydrogenation and methylation reactions, resulting in the formation of cyclopentadiene (C_5_H_6_; *m*/*z* = 66) and methyl cyclopentadiene (C_6_H_8_; *m*/*z* = 80), respectively. Furthermore, they have identified fulvene (C_6_H_6_; *m*/*z* = 78) formed through the dehydrogenation of methyl cyclopentadienes (C_6_H_8_). Fulvene is a precursor for the production of benzene, the first aromatic ring compound. Direct methylation of fulvene, as well as dehydrogenation-methylation of C_6_H_8_, leads to the production of methyl fulvene (C_7_H_8_; *m*/*z* = 92), which is the primary precursor to toluene. This process can continue to generate other alkylated benzenes, including various isomers of xylene and trimethylbenzene. In the current experiment, masses 78 and 92 are detected, while 66 and 80 are not observed [Fig. 2 ▸(*b*)]. To determine the identities of the detected peaks, a comparison between the CIY-PES obtained for masses 78 and 92, together with the relevant reference spectra are presented in Figs. 3 ▸(*e*) and 3 ▸(*f*), respectively (Kimura, 1981 ▸; Cesarini *et al.*, 2022 ▸). The results indicate that benzene and toluene are the parent molecules associated with the detected peaks. However, it was not possible to detect intermediates such as fulvene and methyl fulvene in our experiments. We hypothesize that this limitation could be attributed to the experimental configuration of the reactor used in this study, *i.e.* the second inert zone of the packed bed.

In the present study, two peaks at masses 105 (C_8_H_9_^+^) and 106 (C_8_H_10_^+^) are observed in the TOF spectrum as shown in Fig. 2 ▸(*b*). Considering the reaction mechanism, it is inferred that xylene (C_8_H_10_) is the appropriate parent molecule associated with these peaks. The CIY-PES for mass 106 and the aggregation of CIY-PES for xylenes’ main fragments (*i.e.* masses 106, 105 and 91) are compared with reference spectra in Fig. 3 ▸(*g*). The CIY-PES for mass 106 does not fully coincide with the reference spectra of xylenes (Koenig *et al.*, 1974 ▸; Salaneck, 1981 ▸) (see Fig. S7). However, the aggregation of CIY-PES for masses 106, 105 and 91, presenting electrons coming from three various ionization channels, more closely matches the reference spectra. This aligns with the practice in reference spectra where electrons from all ionization channels (including both inner valence ionization and outermost shell ionization) are accounted for. Conversely, in CIY-PES for mass 106, only electrons produced in the ionization channel of 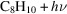

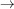

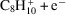
 are taken into consideration. The reference spectra of the xylenes shown in Fig. 3 ▸(*g*) are measured during a separate experiment without the reactor. Fig. 3 ▸(*g*) shows that the effluent stream contains a combination of different isomers of C_8_H_10_, with *m*-xylene appearing to be the most abundant one. Xylene isomerism offers a convenient benchmark problem for quantitative isomer discrimination, described in detail in Section 3.4, which is a novel aspect in PEPICO analysis introduced in our work.

In addition to the various isomers of xylene, isomers of trimethylbenzene can be generated through direct methyl­ation or dehydrogenation-methylation of lighter hydrocarbons (Cesarini *et al.*, 2022 ▸). Despite the detection of only a negligible number of cations with mass 120 in the TOF spectrum [Fig. 2 ▸(*b*)], the comparison of the extracted CIY-PES with reference spectra [Fig. 3 ▸(*h*)] indicates the presence of a mixture of different isomers of trimethylbenzenes in our experiment (Longetti *et al.*, 2020 ▸). It is worthwhile mentioning that CIY-PES for mass 120 only shows the electrons coming from the ionization channel that leads to the removal of one electron. On the other hand, reference PES includes electrons coming from all ionization channels.

### 3.4. Isomer quantification

Quantitative discrimination of isomers from PEPICO data provides a valuable tool for analyzing complex reaction pathways in catalytic reactions of hydrocarbons. The reference spectra for different isomers of xylene, measured in the absence of reactor, are depicted in Fig. 3 ▸(*g*) along with the CIY-PES for mass 106 and its combination with masses 105, and 91 from the reactor effluent stream. The major differences between these spectra lies in their first and third bands in the 8–10 and 13–14 eV regions of the binding energy, respectively. The ionization channel of 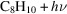

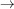

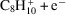
 is correlated to the electrons with binding energy of 8–10 eV. Moreover, for the reference spectra, electrons coming from the background (H_2_O, N_2_ and O_2_) do not overlap with xylene electrons coming from the ionization channel of 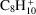. Therefore, we are going to focus on the first band to quantitatively distinguish the ratio of different isomers of xylene in the effluent.

**Figure 4:**
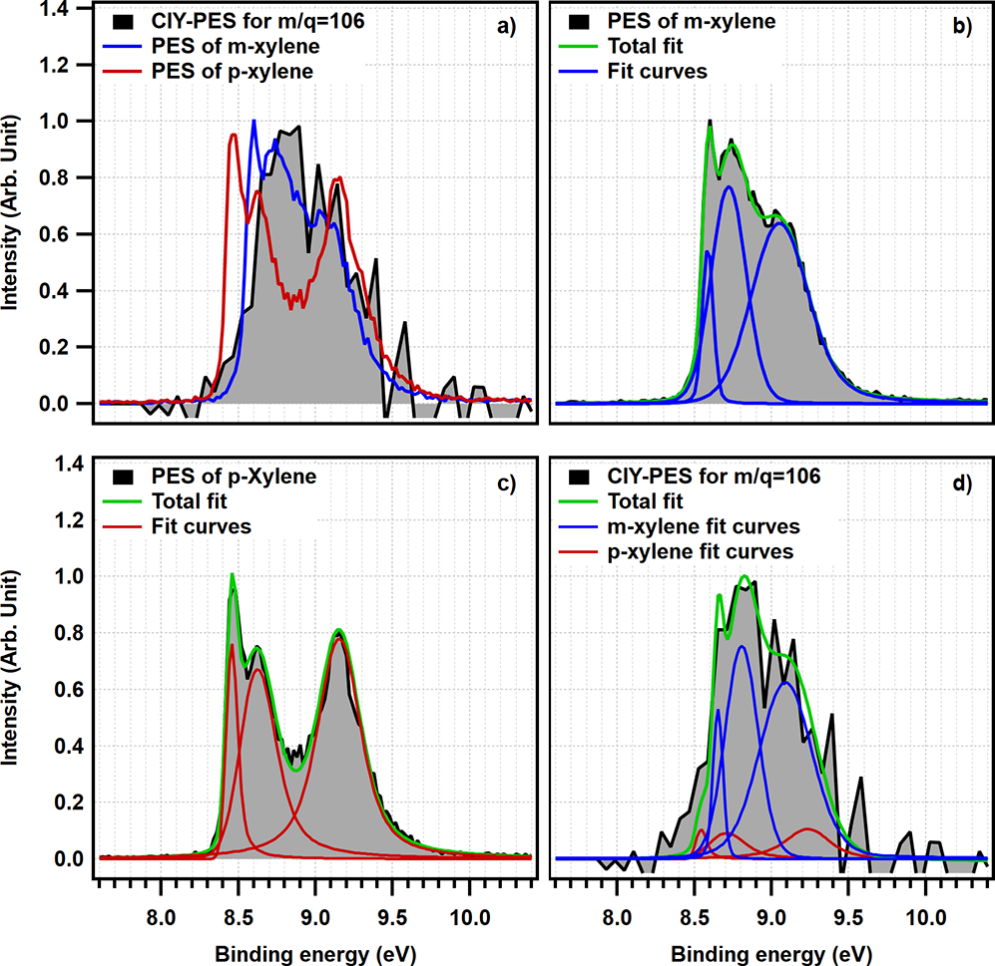
Comparison between (*a*) CIY-PES for mass 106 with reference PES of *m*-xylene and *p*-xylene, (*b*) and (*c*) reference PES of *m*-xylene and *p*-xylene respectively, with their corresponding fits, and (*d*) CIY-PES for mass 106 with the deconvoluted peaks.

A comparison between CIY-PES for mass 106 with reference spectra of xylene isomers is given in Fig. 4 ▸(*a*). The CIY-PES for mass 106 does not have the two separate peaks which are characteristic of *p*-xylene. In addition, its FWHM is almost equal to *m*-xylene. Therefore, *m*-xylene is the dominant isomer among the products. But what is the exact branching ratio?

In order to quantitatively determine the ratio of isomers, each reference spectrum has been deconvoluted using a collection of asymmetrically distorted Voigt profiles. For reference PES of *m*- and *p*-xylene, aggregation of three Voigt profiles is required to adequately describe the collected data [Figs. 4 ▸(*b*) and 4(*c*), respectively],
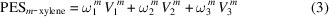

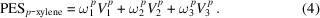
Here, ω shows the individual weight of each Voigt function. Then, these two groups of peaks were mixed in a specific ratio, while the peak shapes, center distances and intensity ratios within each group were fixed according to their values estimated from pure reference compounds. By iteratively adjusting the ratio of these two groups of peaks and minimizing the difference between the generated spectra and the one with unknown mixing ratio, one could quantify the mixing ratio. First, the method was benchmarked against two control mixtures of *m*- and *p*-xylenes with known compositions, 50:50 and 75:25, returning estimates of 56:44 and 77:23, respectively. This outcome demonstrates that, although promising, the isomer quantification analysis requires PEPICO data with higher signal-to-noise ratio than in the present data, and more systematic collection of calibration datasets. Next, we applied the same routine to analyze the reactor effluent. Fig. 4 ▸(*d*) demonstrates the quality of the resulting model fit. Based on this method, and considering the area of peaks, almost 85% of the electrons detected in coincidence with 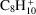 cation are coming from *m*-xylene and the remaining 15% are related to *p*-xylene. This result agrees well with the selectivities reported in the literature for unmodified ZSM-5 (Zhang *et al.*, 2015 ▸; Gao *et al.*, 2020 ▸). Although *p*-xylene is more likely to be the prevalent primary product of toluene methylation inside the micropores, unmodified ZSM-5 in these studies is thought to contain external acid sites that rapidly isomerize *p*-xylene. Unneberg & Kolboe (1988 ▸) have shown that the xylene isomer distribution over ZSM-5 catalysts can shift from *meta* to *para* with increasing time on stream. In our future work, we will apply isomer-selective PEPICO to systematically characterize *p*-/*m*-xylene selectivity in zeolites at low-pressure conditions, *i.e.* in the limit of low DME exposure, to fully understand what controls their intrinsic xylene selectivity.

## 4. Conclusion

*Operando* PEPICO mass-spectrometry is emerging as a valuable analytical tool for investigations of reaction mechanisms and kinetics in heterogeneous catalysis. Currently, the scope of the technique and its adoption in the catalysis research community are constrained by the limited availability of dedicated facilities around the globe. We have established the analysis of catalytic reactor effluents using PEPICO and, potentially, other photoionization-based methods at the FinEstBeAMS beamline at MAX IV Laboratory. This capability was demonstrated using dimethyl ether conversion on a prototypical ZSM-5 catalyst (at 375°C and 5 × 10^−7^ mbar total pressure) as a benchmark reaction, which agreed with the product distribution expected from the literature. Due to the specific configuration of the reactor packing in our proof-of-principle study, we have not observed either ketene or methyl radical, both highly reactive intermediates that were recently revealed by *operando* PEPICO. However, we have quantitatively determined the ratio of xylene isomers in the product stream by deconvoluting their coincidence photoelectron spectra, opening up a new avenue for quantitative isomer-selective PEPICO analysis in kinetic studies of heterogeneously catalyzed reactions.

To enhance the technique, a differential pumping system is developing to facilitate high-pressure studies as well. In the current study, the reactor pressure was in the range 10^−7^ to 10^−6^ mbar. However, with the implementation of the differential pumping system, it will be feasible to conduct PEPICO experiments while the reactor is at atmospheric pressure. Overcoming the constraint of being limited to steady-state conditions, due to prolonged data acquisition times, requires using a different type of electron analyzer, such as electron TOF, which has higher transmission than the hemispherical one.

## Supplementary Material

Supporting Figures S1 to S7. DOI: 10.1107/S1600577524004405/vy5024sup1.pdf

## Figures and Tables

**Table 1:** Catalytic experiments at FinEstBeAMS in the global context

Facility	Beamline	Pressure range	Energy range	Analyzer	Detector	Techniques	Reference
SLS†	VUV	10^−8^–10^3^ mbar	3–150 eV	e-TOF	Position-sensitive DLD	PIMS, iPEPICO, i^2^PEPICO	(Sztáray *et al.*, 2017 ▸; SLS, 2023 ▸)
				ion-TOF	Position-sensitive DLD		

NSRL	BL03U	30–760 Torr	5–21 eV	ion-TOF	Cone-shaped stainless steel anode	PIMS	(Zhou *et al.*, 2016 ▸)

SOLEIL	DESIRS (SAPHIRS endstation)	10^−8^ mbar to a few mTorr	5–40 eV	e-TOF	Position-sensitive detector	i^2^PEPICO	(Tang *et al.*, 2015 ▸)
				ion-TOF	Position-sensitive detector		

MAX IV	FinEstBeAMS (GPES)	10^−8^–10^−6^ mbar‡	4.4–1000 eV	Hemispherical electron energy analyzer	Position-sensitive resistive anode	XPS, PIMS, PEPICO, PIPICO	(Pärna *et al.*, 2017 ▸; Kooser *et al.*, 2020 ▸)
				ion-TOF	Position-sensitive DLD		

† SLS shutdown in 2023 will limit global PEPICO capacity for a few years.

‡ Differentially pumped molecular beam extraction for experiments up to 10^3^ mbar is under design/construction at the University of Oulu.

**Table 2:** Detailed breakdown of ionization fragments at 40 eV photon energy (in coincidence with 7 to 17 eV binding energy), corresponding to the TOF spectrum of true coincidences

Mass (a.m.u.)	Chemical formula	Mass (a.m.u.)	Chemical formula
1	H^+^	39–42	C_3_H_*n*_^+^ (*n* = 3–6)
2	H_2_^+^	44	CO_2_^+^
14	N^+^	50–52	C_4_H_*n*_^+^ (*n* = 2–4)
15–16	CH_*n*_^+^ (*n* = 3–4)	65	C_5_H_5_^+^
17	OH^+^	77–78	C_6_H_*n*_^+^ (*n* = 5–6)
18	H_2_O^+^	91–92	C_7_H_*n*_^+^ (*n* = 7–8)
26–27	C_2_H_*n*_^+^ (*n* = 2–3)	105–106	C_8_H_*n*_^+^ (*n* = 9–10)
28	C_2_H_4_^+^, N_2_^+^, CO^+^	120	C_9_H_12_^+^
29–30	C_2_H_*n*_^+^ (*n* = 5–6)	128	C_10_H_8_^+^
32	O_2_^+^	141–142	C_11_H_*n*_^+^ (*n* = 9–10)
37	C_3_H^+^	156	C_12_H_12_^+^
