# Supplementary material for: PEPICO analysis of catalytic reactor effluents towards quantitative isomer discrimination: DME conversion over a ZSM-5 zeolite
Source: J Synchrotron Radiat. 2024 Jun 25;31(Pt 4):841–50. doi: 10.1107/S1600577524004405 (PMC11226177; doi:10.1107/S1600577524004405)
Supplement: Supplementary file 1 [file s-31-00841-sup1.pdf]

JOURNAL OF SYNCHROTRON RADIATION

**Volume 31 (2024)**

**Supporting information for article:**

# PEPICO analysis of catalytic reactor effluents towards quantitative isomer discrimination: DME conversion over a ZSM-5 zeolite

**Morsal Babayan, Evgeniy Redekop, Esko Kokkonen, Unni Olsbye, Marko Huttula and Samuli Urpelainen**

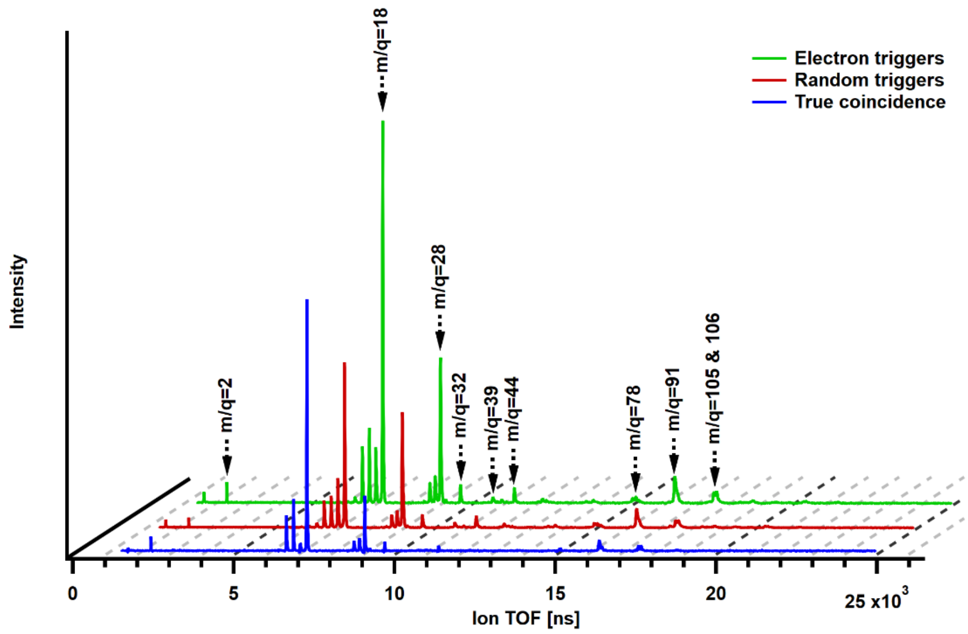


**Figure S1** The TOF mass spectra of ions for random triggers, electron triggers, and true coincidences recorded with photon energy of 40 eV.

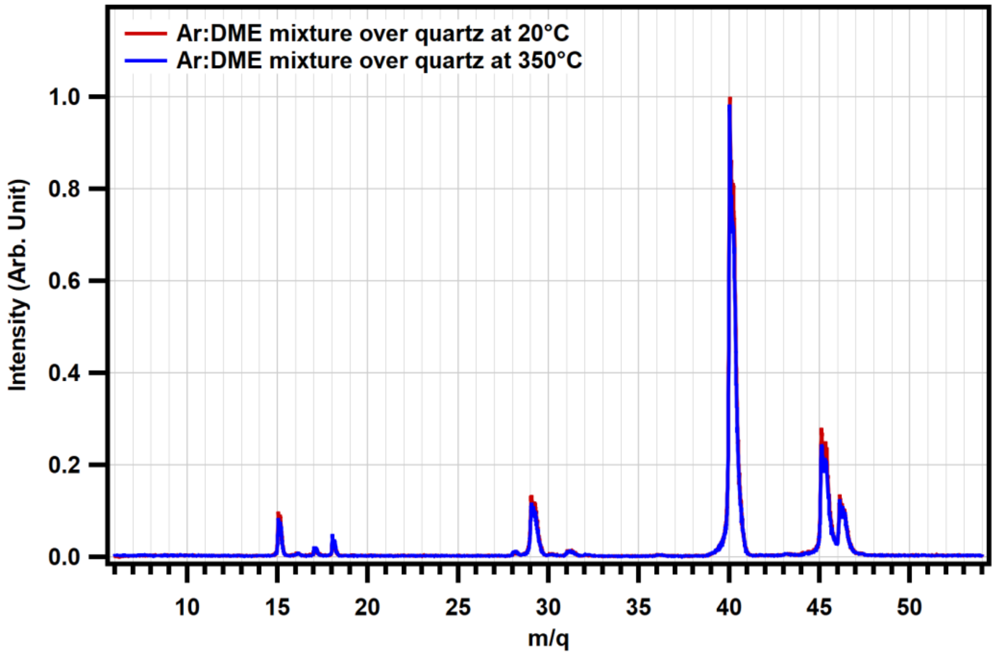


**Figure S2** The TOF mass spectra of a mixture of Ar and DME (3:1 ratio) flowing over the empty reactor at 20 and 350 °C temperatures.

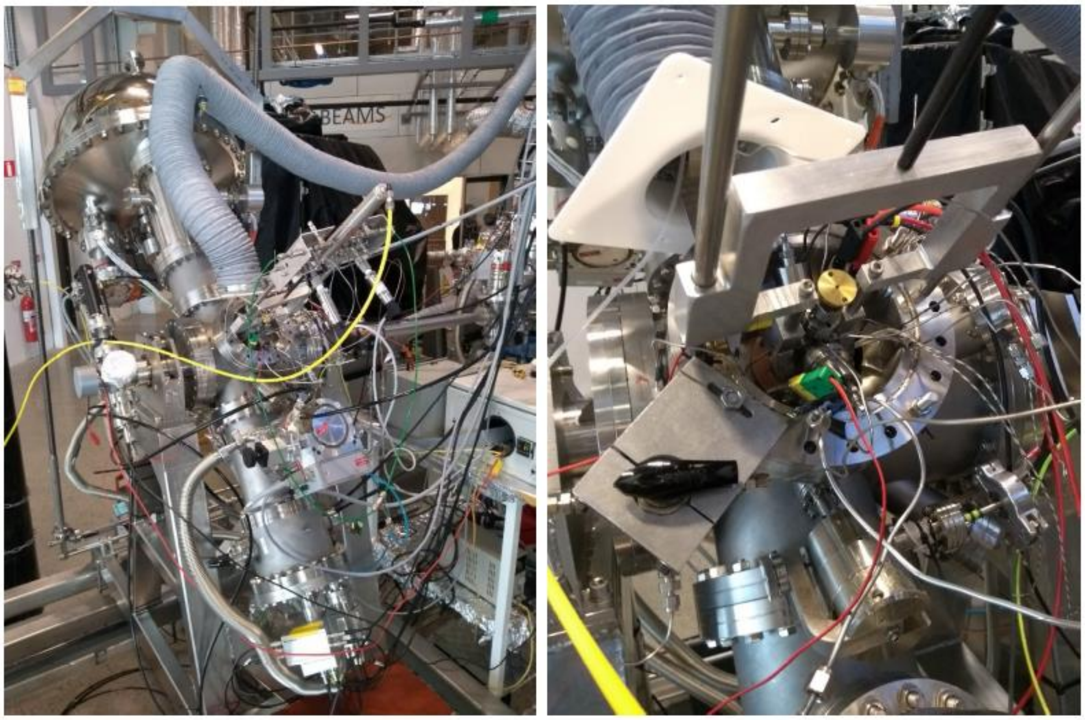

**Figure S3** Photographs of the experimental setup at the GPES.

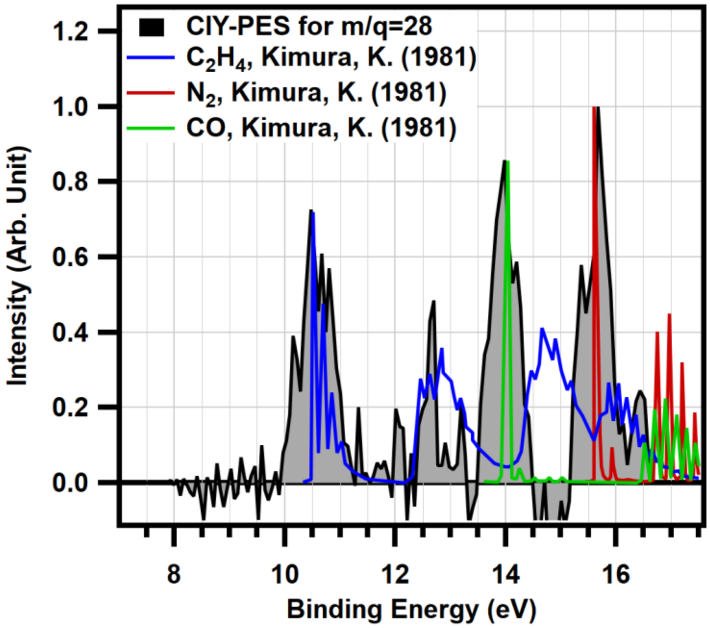


**Figure S4** Comparison of extracted coincidence ion yield PES at 40 eV photon energy with reference spectra for ions with mass 28 (Kimura, 1981).

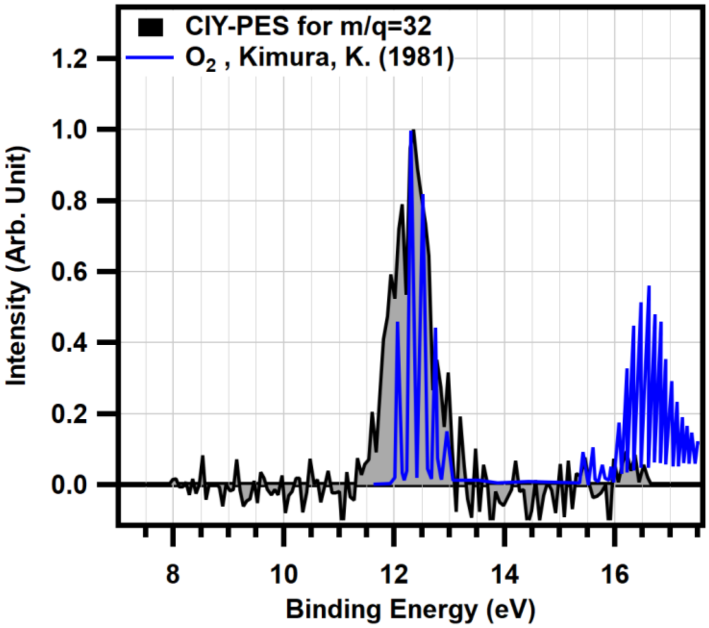


**Figure S5** Comparison of extracted coincidence ion yield PES at 40 eV photon energy with reference spectrum for ions with mass 32 (Kimura, 1981).

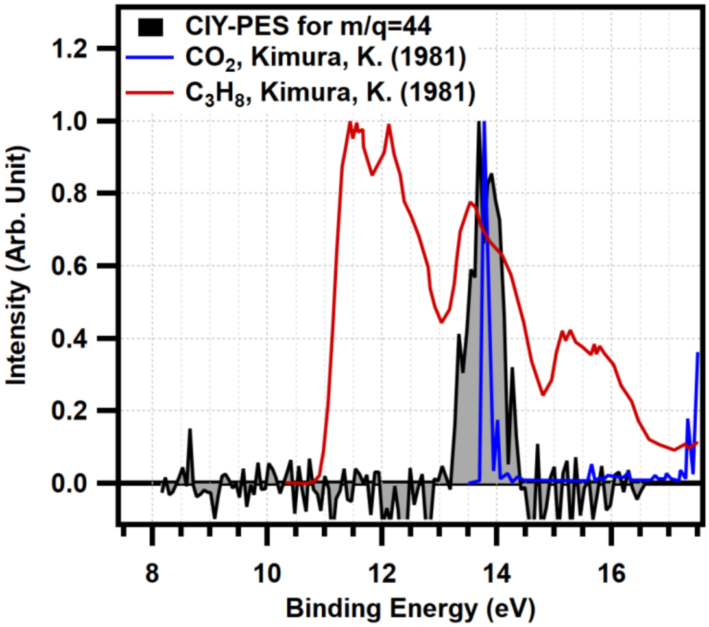


**Figure S6** Comparison of extracted coincidence ion yield PES at 40 eV photon energy with reference spectra for ions with mass 44 (Kimura, 1981).

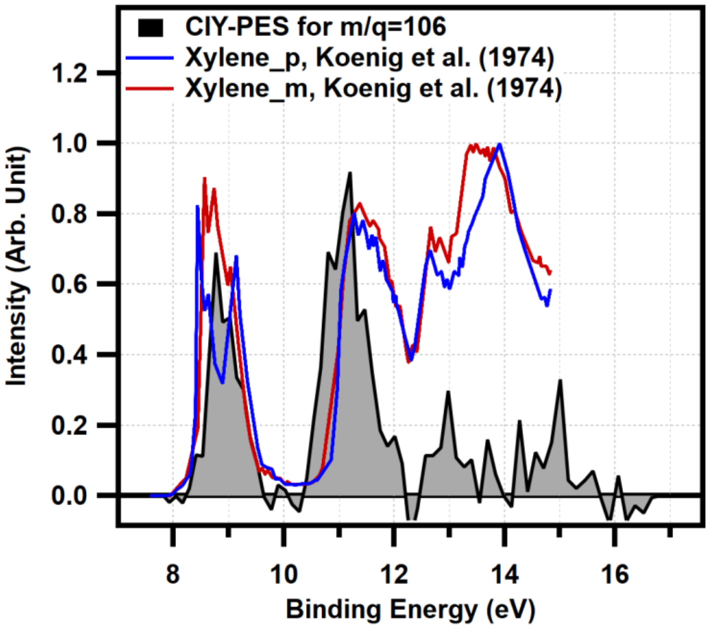


**Figure S7** Comparison of extracted coincidence ion yield PES at 40 eV photon energy with reference spectra for ions with mass 106 (Koenig et al., 1974).

## References

Kimura, K. (1981). *Handbook of HeI Photoelectron Spectra of Fundamental Organic Molecules.* Halsted Press.

Koenig, T., Tuttle, M. & Wielesek, R. (1974). *Tetrahedron Lett.* **15**(29), 2537–2540.
